# Quantitative proteomic profiling reveals sexual dimorphism in the retina and RPE of C57BL6 mice

**DOI:** 10.1186/s13293-024-00645-9

**Published:** 2024-10-30

**Authors:** Geeng-Fu Jang, John S. Crabb, Allison Grenell, Alyson Wolk, Christie Campla, Shiming Luo, Mariya Ali, Bo Hu, Belinda Willard, Bela Anand-Apte

**Affiliations:** 1 Department of Ophthalmic Research, Cole Eye Institute, Cleveland Clinic, Cleveland, OH USA; 2 Department of Pharmacology, Case Western Reserve University, Cleveland, OH USA; 3 Department of Quantitative Health Sciences, Lerner Research Institute, Cleveland Clinic, Cleveland, OH USA; 4 Mass Spectrometry Laboratory for Protein Sequencing, Lerner Research Institute, Cleveland Clinic, Cleveland, OH USA; 5 Department of Ophthalmology, Cleveland Clinic Lerner College of Medicine, Case Western Reserve University, Cleveland, OH USA

## Abstract

**Background:**

Sex as a biological variable is not a common consideration in molecular mechanistic or preclinical studies of retinal diseases. Understanding the sexual dimorphism of adult RPE and retina under physiological conditions is an important first step in improving our understanding of sex-based physio-pathological mechanisms.

**Methods:**

Isobaric tags for relative and absolute quantitation (iTRAQ) were used for quantitative proteomics of male and female mouse retina and RPE (10 mice of each sex for each tissue type). Differentially expressed proteins were subjected to Gene Ontology (GO) analysis and Ingenuity Pathway Analysis (IPA).

**Results:**

Differential expression analysis identified 21 differentially expressed proteins in the retina and 58 differentially expressed proteins in the RPE. Ingenuity pathway analysis identified the top canonical pathways differentially activated in the retina to be calcium transport I, nucleotide excision repair, molecular transport and cell death and survival. In the RPE, the top canonical pathways were calcium signaling, dilated cardiomyopathy signaling, actin cytoskeletal signaling and cellular assembly and organization.

**Conclusions:**

These results provide insights into sex differences in the retina and RPE proteome of mice and begin to shed clues into the sexual dimorphism seen in retinal diseases.

**Supplementary Information:**

The online version contains supplementary material available at 10.1186/s13293-024-00645-9.

## Abstract (plain language summary)

According to the Centers for Disease Control and Prevention (CDC) an estimated 93 million adults in the United States are at high risk for serious vision loss. Besides the devastating effect on the individual’s quality of life, the economic cost of major vision loss is estimated to increase to $373 billion by 2050. In the United States, the Society for Women’s Health Research Women’s Eye Health Working Group established that women are at a higher risk of developing loss of vision due to eye diseases such as age-related macular degeneration, thyroid eye disease or glaucoma than men. The innate biological differences between males and females that contribute to disease pathology are unknown. There are several cell types in the back of the eye that are responsible for sight. The retina contains light sensing cells (photoreceptors) that capture photons and transmits them to the brain along neural networks as both chemical and electric signals for visual perception. The retinal pigment epithelium (RPE) is a layer of cells underneath the retina that is critical for the normal functioning of the retina. We investigated quantitative differences in the protein composition in the retina and RPE between male and female mice. Using stringent analyses, we identified potential biological pathways that are different between male and female tissues that could contribute to disease pathology. These findings begin to provide possible mechanisms for the sexual dimorphism of ocular diseases.

**Supplementary Information:**

The online version contains supplementary material available at 10.1186/s13293-024-00645-9.

## Highlights

- The mouse retina and retinal pigment epithelium (RPE) proteome exhibit significant sex differences at baseline.
- This dataset will serve as a resource for researchers using mouse models to study ocular physiology and pathology.
- These findings encourage scientists evaluating protein alterations in mouse models of ocular disease to include a careful comparison of sex (and possibly age) in their experimental design to improve the reproducibility and appropriate interpretation of experiments.

**Supplementary Information:**

The online version contains supplementary material available at 10.1186/s13293-024-00645-9.

## Introduction

According to the World Health Organization, approximately 2.2 billion people around the globe have vision impairment. Sex-related differences in ocular diseases have been poorly studied despite epidemiological evidence which suggests that sex can modify the risk of developing vision-threatening diseases such as age-related macular degeneration, diabetic retinopathy and glaucoma [1–3]. Sex (male and female) is a significant contributor to many physiological functions distinct from those pertaining exclusively to reproduction. In vertebrate eyes, photoreceptor distribution and M cone polymorphisms result in more sensitive color perception in females [4]. Ocular coherence tomography (OCT) studies have demonstrated the macular retina and choroid to be thicker in men [5]. Differences in retinal structure and function have been seen in aging male and female rats [6]. Functional studies with photopic and scotopic electroretinograms also show sex-based differences [7–10]. A recent study demonstrated that female rd10 mice were more susceptible to retinal degeneration than their male counterparts [11]. In addition, a new report shows that sex has a strong influence on eye and brain metabolism in a tissue and metabolic state-specific manner [12]. The molecular mechanism(s) that underly these sexually dimorphic changes are currently unknown. Improving our understanding of these mechanisms will be critical for developing therapeutics for prevention and treatment in a sex- specific manner.

To understand the origin of this sexual dimorphism in normal eyes, we reasoned that it was important to identify quantitative differences in proteins in the RPE and retina of male and female adult mice. To this end we performed quantitative proteomics using LC MS/MS iTRAQ technology and identified differentially expressed proteins in the retina and RPE of male and female C57BL6 wild-type mice.

## Materials and methods

### Animals

C57 BL6/J wild-type mice 12 weeks of age (n = 10 males and n = 10 females) were purchased from Jackson Laboratory (Bar Harbor, ME) and housed in the Cole Eye Institute vivarium under a 12-h light/dark cycle. All animal experiments were approved by the Cleveland Clinic Institutional Animal Care and Use Committee (IACUC) and conducted in strict accordance with the National Institutes of Health Guidelines for the Care and Use of Animals in Research.

### Preparation of retina and RPE protein lysates

Following euthanasia (cervical dislocation), eyes were immediately enucleated and kept on ice. The anterior segment and lens were removed and the retina from each eye carefully peeled off and snap frozen on dry ice in separate tubes. Retina from the left eye of each mouse was used for analysis. The posterior segments (RPE/choroid/sclera) from both eyes of a mouse were cut to flatten, placed in a microfuge tube with 200 μL of proteomics lysis buffer (100 mM TEAB, 2% SDS, 1 mM DTT) on ice for 1 h. Vigorous tapping of the tube allowed for dissociation of the RPE from the choroid/Bruch’s membrane/sclera as described previously [13, 14]. The choroid/sclera were removed from the tube and the RPE cells were lysed by pipetting through a 26–27-gauge needle 15–20 times. The lysates were spun down in a microcentrifuge at 14000 rpm for 10 min at 4 ℃ and the supernatant was used for analysis. We have previously demonstrated that this method allows for good separation of retina and RPE. However, we cannot discount possible contamination of the RPE preparations with small amounts of choroid.

### Sample preparation

Each retina tissue fraction was individually homogenized in 100 mM TEAB, 2% SDS, and 2 mM DTT, centrifuged to remove insoluble material and the protein concentration of the soluble protein fraction estimated by the Bicinchoninic Acid assay. Subsequently, each fraction was reduced with 10 mM DTT at room temperature, alkylated with 40 mM iodoacetamide for 1 h, and then quenched with 40 mM DTT [15]. Following reduction and alkylation, protein was precipitated with two volumes of ice-cold acetone. The protein pellets were resuspended in 100 mM TEAB buffer containing 0.5 mM CaCl2 and were digested overnight at 37 ℃ with trypsin (initially with 2% trypsin (w/w) for 2 h, followed with fresh 2% (w/w) for 15 h, and then 1% (w/w) for 2 h the next day). Following proteolysis, soluble peptides were quantified by AccQ-Tag amino acid analysis [16, 17]. Gender-specific pooled reference samples were prepared for proteomic analyses by combining equal amounts of proteolyzed protein from 10 male and 10 female retinas (10 μg/specimen) and from 8 male and 8 female RPE (5 μg/specimen).

### ITRAQ labeling and peptide fractionation

iTRAQ labeling with an 8-plex iTRAQ kit was performed as previously described [17–21]. Tryptic digests of the individual retina and RPE samples and the pooled reference samples were each labeled with a single iTRAQ tag and combined in a total of 6 iTRAQ labeled batches as follows. Batch 1 contained the pooled male retina reference sample and 7 individual female retina samples; Batch 2 contained the pooled female retina reference sample and 7 individual male retina samples. Batch 3 contained with the pooled female retina and the pooled male retina reference samples, 3 individual male retina and 3 individual female retina samples. Batch 4 contained the pooled male RPE reference sample and 7 individual female RPE samples. Batch 5 contained the pooled female RPE reference sample and 7 individual male RPE samples. Batch 6 contained the pooled female RPE and pooled male RPE reference samples, 3 individual male RPE and 3 individual female RPE samples (*Supplementary Fig. 1 for experimental design). Equal amounts of each tryptic digest (25 µg per retina pooled reference and individual samples and 19 µg per RPE reference and individual samples) were used for labeling with a unique 8-plex iTRAQ tag, then the labeled peptides were combined in the above batches and dried for subsequent HPLC fractionation. Each dried batch was resuspended and individually fractionated by reverse-phase high performance liquid chromatography (RPHPLC) at pH 10 on an Agilent Zorbax 300 Extend C18 column (3.5 µ particle size, 2.1 × 150 mm). Chromatography was performed at a flow rate of 200 µL/min using 5 mM NH_4_OH/aqueous acetonitrile solvents, a 0.6%/min acetonitrile gradient over 45 min, a 1.3% acetonitrile gradient over another 10 min, and finally 90% acetonitrile over 5 min; absorbance was monitored at 214 nm. Chromatography fractions encompassing the entire elution were selectively combined, dried, resuspended in 2% formic acid, and filtered with a 0.22 μ filter (Sigma) prior to LC–MS/MS. A total of 9 chromatography fractions per batch were analyzed by LC–MS/MS using an Orbitrap Exploris 480 mass spectrometer (ThermoScientific).

### LC–MS/MS method

Exploris was equipped with a Vanquish Neo UHPLC system, a trapping column (PepMap Neo C18, 5 mm × 30 µm id, 5 μm particle size) and a C18 capillary column (Easy Spray PepMap Neo C18, 50 cm × 75 µm id, 2 μm particle size, 100 Å pore size). Each fraction (10 μL) was injected, trapped and washed in the trapping column at 5 μL/min with 2% B for 5 min and then eluted at a flow rate of 0.3 μL/min using mobile phase A (0.1% formic acid in H_2_O) and B (0.1% formic acid in acetonitrile). The gradient was held at 2% B for 5 min, % B was increased linearly to 44% in 105 min, increased linearly to 99% B in 10 min, and maintained at 99% B for 6 min. The samples were analyzed using a data-dependent acquisition method which involved full MS1 scans from 350 to 1500 Da in the Orbitrap MS at a resolution of 120,000 (profile). This was followed by HCD (0.7 Da isolation window) on precursors (charge states of 2 to 5) at 36% nCE and orbitrap detection at a resolution of 45,000 (profile) with the first mass 107 Da. MS/MS spectra were acquired for 3 s following one full MS1 scan. Dynamic exclusion was enabled with 1 repeat where ions within 10 ppm were excluded for 30 s.

### Protein identification

Protein identification utilized the Mascot 2.7 search engine and the UniProtKB/Swiss-Prot database (version 2022_04, 17,138 mouse sequences). The raw data generated from Exploris were converted to MGF files using Mascot Distiller 2.8.2. Database search parameters were restricted to three missed tryptic cleavage sites, a precursor ion mass tolerance of 10 ppm, a fragment ion mass tolerance of 20 mmu, and a false discovery rate of ≤ 1%. Protein identification required the detection of a minimum of two unique peptides per protein. Fixed protein modifications included N-terminal and ε-Lys iTRAQ modifications and S-carbamidomethyl-Cys. Variable protein modifications included Met oxidation, Asn and Gln deamidation, and iTRAQ Tyr. A minimum Mascot ion score of 20 was used for accepting the peptide MS/MS spectra.

### Protein quantitation

The iTRAQ tag intensities on individual male and female mouse peptides versus their opposite gender pooled reference samples were quantified by the weighted average (ratio of the summed intensities) method [22] using the Mascot 2.7 Summed Intensities Program. Protein quantitation required a minimum of two unique peptides per protein, utilized a reporter ion tolerance of 10 ppm, and a maximum Expect value of 0.05. Protein ratio calculations were determined in log space and were transformed back to linear ratios for reporting.

### Statistical analysis

Limma (Linear Models for Microarray Data) package in R was used to normalize the mass spectrometry iTRAQ proteomics data. After normalization, no significant batch effects were detected. Means and standard error of the mean (SEM) were calculated for proteins quantified. Differential expression (DE) analyses were performed with limma and the results were adjusted for multiple-testing using the Benjamini–Hochberg procedure [9, 10]. Gender differences were sought by evaluating quantitative differences between the proteomic results from the individual male and female specimens relative to the pooled reference samples from the opposite gender. Only proteins showing significant difference in at least 7 of the 10 Male vs FemalePool and 7 of the 10 Female vs MalePool were considered. The following three criteria were used for selecting possible gender differences between male and female mouse retina and RPE with the overriding requirement that specific proteins elevated in one gender were decreased in the opposite gender. (1) A fold change (FC) ≥ 1 standard deviation (SD) from the mean in both male and female tissues with adjusted *p*-values ≤ 0.05; (2) A FC not restricted by SD from the mean in both male and female tissue with adjusted *p*-values ≤ 0.05; (3) A FC ≥ 2 SD with adjusted *p-*values ≤ 0.05 in one gender but no significant FC in the opposite gender.

### Bioinformatics

Bioinformatic analyses were performed with Ingenuity Pathway Analysis (IPA) (Qiagen) and over-representation analysis (ORA) with g:Profiler [23]. For IPA, significant proteins that were differentially expressed in male and female retina and RPE were uploaded onto Qiagen IPA for Core analysis and then overlaid with the global molecular network in the Ingenuity pathway knowledge base. Significant canonical pathways, disease and function, regulator effects, upstream regulators and gene networks were analyzed. For ORA the significant proteins were entered into g:GOSt, Functional Profiling module (https://biit.cs.ut.ee/gprofiler/gost last accessed on July 9th, 2024). The settings used were (a) Organism: *Mus musculus* (b) Statistical domain scope: All known genes (c) Significance threshold: Benjamini–Hochberg FDR (d) User threshols:0.05 and (e) Numeric IDs treated as: ENTREZGENE_ACC. Data sources selected included the Gene Ontology for Molecular Function (GO-MF), Cellular Component (GO-CC), and Biological Process (GO-BP). We excluded electronic GO annotations. In addition, we analyzed the Kyoto Encyclopedia of Genes and Genomes (KEGG), Reactome (REAC) and Wikipathways (WP). Term size was limited to 4000 for specificity.

Bubble charts were generated with Flourish Studio (https://flourish.studio/).

## Results

### Protein profile characterization of pooled male and female mouse retina and RPE

**Fig. 1:**
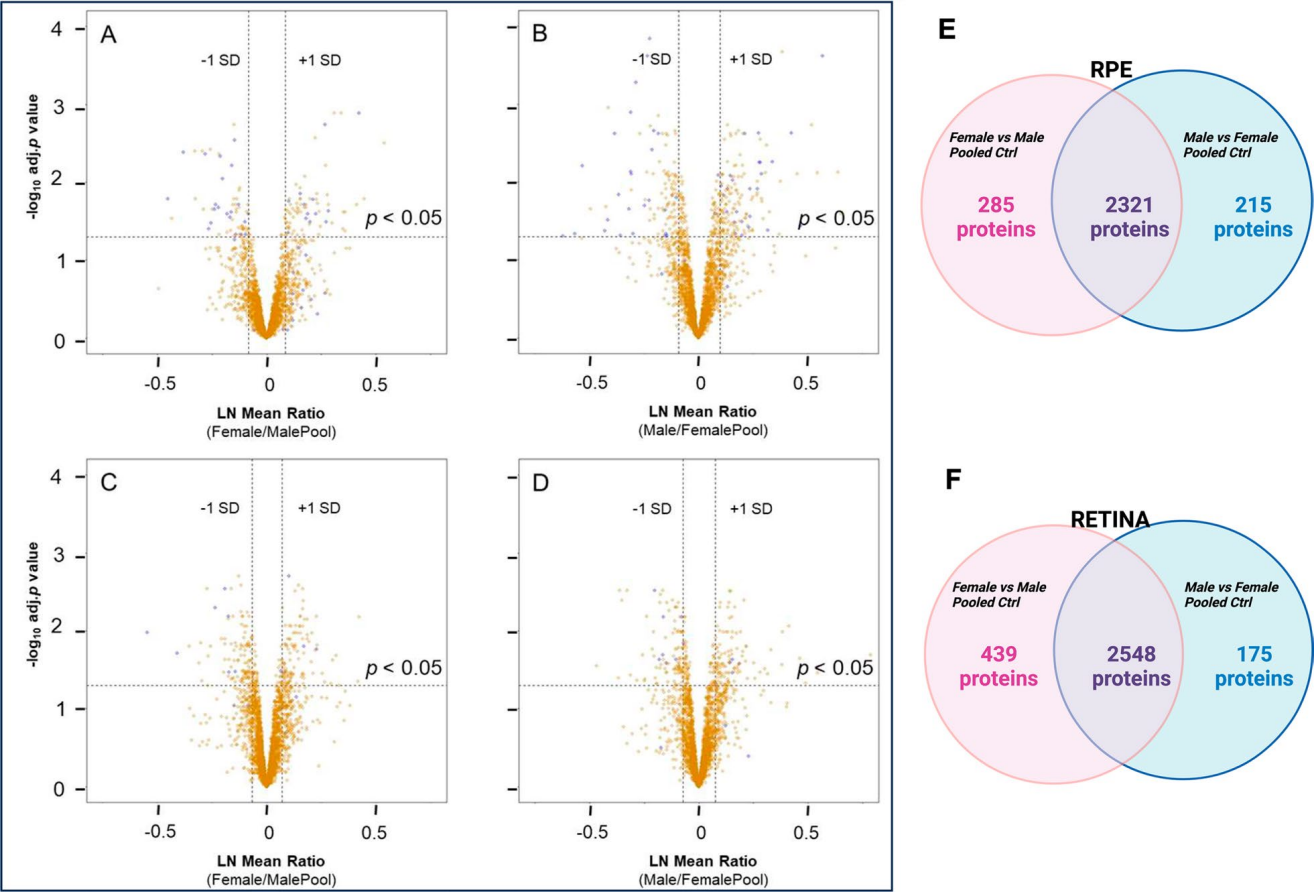
**Quantitative proteomics of mouse RPE and Retina.** **A**–**D** Volcano Plots. Blue represents potential gender differences based on criteria described under Methods and gold represents all other proteins not satisfying criteria. **E**–**F** Venn diagram showing unique and overlapping proteins prior to using criteria for analysis. **A** Volcano plot for 2606 RPE Female vs MalePool; 58 potential gender differences. **B** Volcano plot for 2536 RPE Male vs FemalePool; 58 potential gender differences. **C** Volcano plot for 2987 Retina Female vs MalePool; 21 potential gender differences. **D** Volcano plot for 2723 Retina Male vs FemalePool; 21 potential gender differences. **E** Venn diagram for overlapping and unique proteins in RPE analysis. **F** Venn diagram for overlapping and unique proteins in Retina analysis

In the mouse retina and RPE we quantified > 2700 and > 2500 protein IDs respectively between male and female groups. Distribution of LN mean protein ratios were calculated for Female/MalePool RPE (Fig. 1A), Male/FemalePool RPE (Fig. 1B), Female/MalePool retina (Fig. 1C) and Male/FemalePool retina (Fig. 1D). Distribution of overlapping and unique proteins prior to the application of significance criteria are depicted for RPE (Fig. 1E) and retina (Fig. 1F).Over 80% of all proteins quantified in the RPE and retina were identified in both the Female vs MalePool control and Male vs FemalePool control.

### Identification of differentially expressed (DE) proteins in the retina of male and female C57BL6 mice

Differentially expressed (DE) proteins were identified using an unbiased proteomics approach and statistical analysis comparing the average protein ratios of individual female retinas (n = 10) to the common male pooled retina sample (reference dataset). The results were compared with ratios obtained from individual male retinas (n = 10) to the common female pooled retina samples (reference dataset) (Table 1). Stringent criteria to identify DE proteins included a minimum fold change of one standard deviation from the mean in addition to an adjusted *p*-value of  ≤ 0.05. To be considered as significant, all DE proteins had to be detected in at least 7 of the 10 samples, and the changes had to be reciprocal in both groups i.e., female vs male pool and male vs female pool. A total of 21 DE proteins were identified from a total of 2987 proteins in the Female/Male pool and 2723 proteins in the Male/Female pool. Of the 21 DE proteins, 10 proteins were upregulated, and 11 proteins were downregulated in the female retina compared to male.

**Table 1:** Potential Proteomic Gender Differences in Mouse Retina

UniProt accession	Gene name	Chromosome	Protein	Linear ratio female/male pool (n=2987 proteins)	SEM	Adjusted *p* Value	Sample frequency	Linear ratio male/female pool (n=2723 proteins)	SEM	Adjusted *p* Value	Sample frequency
Q9CQQ8	Lsm7	Chromosome 10	U6 snRNA-associated Sm-like protein LSm7	**1.256**	0.055	1.74E-02	10	***0*.*954***	0.048	4.86E-01	10
P30115	Gsta3	Chromosome 1	Glutathione S-transferase A3	**1.185**	0.035	1.58E-02	7	***0*.*840***	0.130	3.17E-01	7
Q8R1F6	Hid1	Chromosome 11	Protein HID1	**1.146**	0.047	7.06E-02	7	***0*.*834***	0.043	2.33E-02	7
P17563	Selenbp1	Chromosome 3	Methanethiol oxidase	**1.106**	0.007	2.00E-03	10	***0*.*931***	0.013	1.43E-02	10
P01942	Hba	Unplaced	Hemoglobin subunit alpha	**1.088**	0.047	2.05E-01	10	***0*.*816***	0.027	3.06E-03	10
Q6Q477	Atp2b4	Chromosome 1	Plasma membrane calcium-transporting ATPase 4	**1.086**	0.021	3.38E-02	10	***0*.*925***	0.011	6.64E-03	10
Q8BIW1	Prune1	Chromosome 3	Exopolyphosphatase PRUNE1	**1.076**	0.026	9.69E-02	7	***0*.*851***	0.024	6.64E-03	7
Q62383	Supt6h	Chromosome 11	Transcription elongation factor SPT6	**1.067**	0.015	3.27E-02	10	***0*.*901***	0.026	2.33E-02	10
Q80X95	Rraga	Chromosome 4	Ras-related GTP-binding protein A	**1.028**	0.021	4.21E-01	7	***0*.*848***	0.037	2.04E-02	7
O70591	Pfdn2	Chromosome 1	Prefoldin subunit 2	**1.009**	0.013	7.26E-01	10	***0*.*862***	0.039	2.60E-02	10
Q9CQ71	Rpa3	Chromosome 6	Replication protein A 14 kDa subunit	***0.987***	0.035	8.20E-01	10	**1*.*168**	0.046	3.45E-02	10
Q8CFI7	Polr2b	Chromosome 5	DNA-directed RNA polymerase II subunit RPB2	***0.939***	0.015	3.59E-02	10	**1*.*204**	0.050	2.63E-02	10
Q9EPK7	Xpo7	Chromosome 14	Exportin-7	***0.917***	0.033	9.09E-02	10	**1*.*167**	0.040	2.33E-02	10
Q99M31	Hspa14	Chromosome 2	Heat shock 70 kDa protein 14	***0.865***	0.037	3.37E-02	7	**1*.*023**	0.052	7.66E-01	7
Q8QZZ7	Tprkb	Chromosome 6	EKC/KEOPS complex subunit Tprkb	***0.862***	0.058	9.12E-02	8	**1*.*154**	0.034	2.53E-02	7
Q8BTY1	Kyat1	Chromosome 2	Kynurenine--oxoglutarate transaminase 1	***0.841***	0.045	3.41E-02	7	**1*.*040**	0.036	4.51E-01	7
Q78JE5	Fbxo22	Chromosome 9	F-box only protein 22	***0.838***	0.031	6.58E-03	10	**1*.*083**	0.025	4.83E-02	10
Q9DBG7	Srpra	Chromosome 9	Signal recognition particle receptor subunit alpha	***0.826***	0.020	2.87E-03	7	**1*.*042**	0.021	2.34E-01	7
Q9QX60	Dguok	Chromosome 6	Deoxyguanosine kinase, mitochondrial	***0.790***	0.031	5.07E-03	7	**1*.*048**	0.026	2.37E-01	7
P02535	Krt10	Unplaced	Keratin, type I cytoskeletal 10	***0.663***	0.103	1.93E-02	10	**1*.*130**	0.062	1.65E-01	10
Q8R429	Atp2a1	Chromosome 7	Sarcoplasmic/endoplasmic reticulum calcium ATPase 1	***0.578***	0.114	1.05E-02	10	**1*.*255**	0.200	4.08E-01	10

### Identification of differentially expressed (DE) proteins in the RPE of male and female C57BL6 mice

DE proteins in the RPE of male and female mice were identified using the same strategy/criteria as that described above for retina. A total of 58 DE proteins were identified from a total of 2606 proteins in the Female/Male pool and 2536 proteins in the Male/Female pool. Of the 58 proteins, 28 were upregulated and 30 were downregulated in the female RPE compared to the male (Table 2).

**Table 2:** **Potential Proteomic Gender Differences in Mouse RPE**

UniProt accession	Gene name	Chromosome	Protein	Linear ratio female/male pool (n=2606 proteins)	SEM	Adjusted *p* Value	Sample frequency	Linear ratio male/female pool (n=2536 proteins)	SEM	adjusted *p* Value	Sample frequency
A2AQP0	Myh7b	Chromosome 2	Myosin-7B	**1.526**	0.045	1.30E-03	7	***0.860***	0.051	4.92E-02	8
O09161	Casq2	Chromosome 3	Calsequestrin-2	**1.327**	0.071	2.35E-02	10	***0.681***	0.065	2.39E-03	10
Q62082	Myl10	Chromosome 5	Myosin regulatory light chain 10	**1.312**	0.074	3.22E-02	10	***0.587***	0.109	6.18E-03	10
Q61597	Crygc	Chromosome 1	Gamma-crystallin C	**1.308**	0.152	2.68E-01	10	***0.567***	0.199	4.63E-02	10
Q9JJW5	Myoz2	Chromosome 3	Myozenin-2	**1.308**	0.037	1.81E-03	10	***0.866***	0.049	4.88E-02	10
P24622	Cryaa	Chromosome 17	Alpha-crystallin A chain	**1.262**	0.211	4.94E-01	10	***0.536***	0.223	4.92E-02	10
Q7TQF7	Amph	Chromosome 13	Amphiphysin	**1.252**	0.053	2.54E-02	7	***0.845***	0.089	1.51E-01	7
P51667	Myl2	Chromosome 5	Myosin regulatory light chain 2, ventricular/cardiac muscle isoform	**1.238**	0.040	1.67E-02	7	***0.782***	0.064	2.01E-02	7
P04247	Mb	Chromosome 15	Myoglobin	**1.225**	0.130	3.23E-01	10	***0.694***	0.093	1.44E-02	10
P13541	Myh3	Chromosome 11	Myosin-3	**1.219**	0.053	3.22E-02	10	***0.733***	0.065	7.41E-03	10
Q9CY73	Mrpl44	Chromosome 1	39S ribosomal protein L44, mitochondrial	**1.209**	0.043	2.54E-02	7	***0.858***	0.038	2.31E-02	7
Q68FL6	Mars1	Chromosome 10	Methionine--tRNA ligase, cytoplasmic	**1.199**	0.047	2.92E-02	10	***0.939***	0.025	1.02E-01	10
Q9QWL7	Krt17	Chromosome 11	Keratin, type I cytoskeletal 17	**1.198**	0.065	8.63E-02	10	***0.814***	0.031	2.20E-03	10
Q80XN0	Bdh1	Chromosome 16	D-beta-hydroxybutyrate dehydrogenase, mitochondrial	**1.189**	0.031	1.92E-02	7	***0.902***	0.016	1.59E-02	7
P04344	Crygb	Chromosome 1	Gamma-crystallin B	**1.184**	0.089	2.43E-01	10	***0.742***	0.103	4.61E-02	10
P19123	Tnnc1	Chromosome 14	Troponin C, slow skeletal and cardiac muscles	**1.174**	0.120	4.04E-01	10	***0.733***	0.043	1.28E-03	10
P23953	Ces1c	Chromosome 8	Carboxylesterase 1C	**1.167**	0.029	1.39E-02	10	***0.833***	0.029	2.39E-03	10
Q62009	Postn	Chromosome 3	Periostin	**1.160**	0.049	6.97E-02	10	***0.800***	0.017	1.44E-04	10
Q5SX40	Myh1	Chromosome 11	Myosin-1	**1.129**	0.050	1.45E-01	10	***0.809***	0.038	3.84E-03	10
Q9WVJ5	Crybb1	Chromosome 5	Beta-crystallin B1	**1.129**	0.134	5.89E-01	10	***0.699***	0.127	4.88E-02	10
Q8CI43	Myl6b	Chromosome 10	Myosin light chain 6B	**1.129**	0.067	2.69E-01	10	***0.751***	0.034	5.24E-04	10
Q8CIX8	Lgsn	Chromosome 1	Lengsin	**1.124**	0.080	3.66E-01	10	***0.728***	0.090	2.21E-02	10
Q9WUZ5	Tnni1	Chromosome 1	Troponin I, slow skeletal muscle	**1.112**	0.039	1.13E-01	10	***0.799***	0.059	1.72E-02	10
Q62167	Ddx3x	Chromosome X	ATP-dependent RNA helicase DDX3X	**1.108**	0.017	1.74E-02	10	***0.792***	0.023	2.40E-04	10
P82198	Tgfbi	Chromosome 13	Transforming growth factor-beta-induced protein ig-h3	**1.103**	0.025	4.48E-02	10	***0.860***	0.051	4.63E-02	10
P02525	Cryba1	Unplaced	Beta-crystallin A1	**1.102**	0.205	7.94E-01	10	***0.651***	0.146	4.26E-02	10
P62696	Crybb2	Chromosome 5	Beta-crystallin B2	**1.078**	0.145	7.73E-01	10	***0.659***	0.130	3.06E-02	10
Q03740	Cryge	Chromosome 1	Gamma-crystallin E	**1.023**	0.048	8.07E-01	10	***0.736***	0.064	7.92E-03	9
Q3UNZ8	Cryzl2	Chromosome 1	Quinone oxidoreductase-like protein 2	***0.999***	0.027	9.83E-01	10	**1.597**	0.153	3.67E-02	10
Q9EPR5	Sorcs2	Chromosome 5	VPS10 domain-containing receptor SorCS2	***0.994***	0.031	9.37E-01	7	**1.258**	0.072	3.83E-02	7
P59114	Pcif1	Chromosome 2	mRNA (2~-O-methyladenosine-N(6)-)-methyltransferase	***0.992***	0.024	9.06E-01	7	**1.530**	0.060	2.34E-03	7
Q91WD8	Slc24a1	Chromosome 9	Sodium/potassium/calcium exchanger 1	***0.989***	0.036	8.95E-01	7	**1.219**	0.058	3.23E-02	7
P70302	Stim1	Chromosome 7	Stromal interaction molecule 1	***0.964***	0.025	4.39E-01	10	**1.319**	0.054	5.59E-03	10
Q8CGY8	Ogt	Chromosome X	UDP-N-acetylglucosamine--peptide N-acetylglucosaminyltransferase 110 kDa subunit	***0.953***	0.015	2.69E-01	7	**1.311**	0.060	1.22E-02	7
Q4VA53	Pds5b	Chromosome 5	Sister chromatid cohesion protein PDS5 homolog B	***0.947***	0.109	7.84E-01	10	**1*.*266**	0.071	2.77E-02	10
P97298	Serpinf1	Chromosome 11	Pigment epithelium-derived factor	***0.919***	0.015	2.54E-02	10	**1*.*081**	0.018	2.52E-02	10
P47758	Srprb	Chromosome 9	Signal recognition particle receptor subunit beta	***0.908***	0.017	4.68E-02	7	**1*.*179**	0.047	3.29E-02	7
Q61398	Pcolce	Chromosome 5	Procollagen C-endopeptidase enhancer 1	***0.905***	0.022	3.22E-02	10	**1*.*096**	0.015	8.21E-03	10
Q80WW9	Ddrgk1	Chromosome 2	DDRGK domain-containing protein 1	***0.889***	0.026	4.67E-02	7	**1.402**	0.059	5.55E-03	7
P29788	Vtn	Chromosome 11	Vitronectin	***0.879***	0.026	1.95E-02	10	**1*.*120**	0.026	1.48E-02	10
P97434	Mprip	Chromosome 11	Myosin phosphatase Rho-interacting protein	***0.868***	0.036	4.71E-02	7	**1*.*171**	0.028	9.53E-03	7
Q62448	Eif4g2	Unplaced	Eukaryotic translation initiation factor 4 gamma 2	***0.864***	0.017	2.91E-03	10	**1*.*110**	0.021	1.13E-02	10
P21981	Tgm2	Chromosome 2	Protein-glutamine gamma-glutamyltransferase 2	***0.863***	0.044	5.48E-02	10	**1*.*334**	0.062	7.92E-03	10
Q91X72	Hpx	Chromosome 7	Hemopexin	***0.853***	0.026	6.06E-03	10	**1*.*191**	0.050	2.43E-02	10
Q5XKE0	Mybpc2	Chromosome 7	Myosin-binding protein C, fast-type	***0.849***	0.039	2.41E-02	10	**1*.*158**	0.030	8.32E-03	10
O35367	Kera	Chromosome 10	Keratocan	***0.846***	0.043	2.83E-02	10	**1*.*185**	0.058	4.61E-02	10
Q5FW53	Mybphl	Chromosome 3	Myosin-binding protein H-like	***0.834***	0.045	2.54E-02	10	**1*.*142**	0.029	1.22E-02	10
Q61495	Dsg1a	Chromosome 18	Desmoglein-1-alpha	***0.817***	0.029	8.95E-03	7	**1*.*375**	0.104	4.14E-02	7
P11087	Col1a1	Chromosome 11	Collagen alpha-1(I) chain	***0.809***	0.035	4.64E-03	10	**1*.*140**	0.036	2.41E-02	10
Q8CHC4	Synj1	Unplaced	Synaptojanin-1	***0.803***	0.052	2.11E-02	10	**1*.*107**	0.046	1.18E-01	10
Q5SX39	Myh4	Chromosome 11	Myosin-4	***0.792***	0.054	1.95E-02	10	**1*.*316**	0.044	2.34E-03	10
P09470	Ace	Chromosome 11	Angiotensin-converting enzyme	***0.787***	0.058	2.21E-02	10	**1*.*237**	0.034	2.39E-03	10
P07309	Ttr	Chromosome 18	Transthyretin	***0.775***	0.070	3.34E-02	10	**1*.*220**	0.069	4.82E-02	10
P02468	Lamc1	Unplaced	Laminin subunit gamma-1	***0.768***	0.073	3.97E-02	7	**1*.*141**	0.083	2.21E-01	7
P07759	Serpina3k	Unplaced	Serine protease inhibitor A3K	***0.755***	0.048	4.36E-03	10	**1*.*329**	0.056	5.59E-03	10
P07758	Serpina1a	Chromosome 12	Alpha-1-antitrypsin 1-1	***0.682***	0.066	4.09E-03	10	**1*.*266**	0.056	1.23E-02	10
P28665	Mug1	Chromosome 6	Murinoglobulin-1	***0.635***	0.100	1.63E-02	10	**1*.*009**	0.050	9.12E-01	10
Q00898	Serpina1e	Chromosome 12	Alpha-1-antitrypsin 1-5	***0.401***	0.147	2.69E-03	10	**1*.*766**	0.066	2.40E-04	10

### Differentially activated biological pathways in the retina and RPE of male and female C57BL6 mice

Using Ingenuity Pathway Analysis (IPA) we examined the relationship between DE proteins to identify the most significant canonical pathways, upstream regulators and molecular and cellular functions that were differentially activated under baseline conditions in the retina and RPE. Table 3 describes the top canonical pathways differentially activated in the retina to be calcium transport I, nucleotide excision repair, platelet homeostasis, nucleotide excision repair and cellular response to heat stress. Top molecular and cellular functions identified in the retina include molecular transport, cell death and survival, cell cycle, cellular development and DNA replication, recombination and repair. In the RPE (Table 4) the top canonical pathways were calcium signaling, dilated cardiomyopathy signaling, striated muscle contraction, actin cytoskeletal signaling and hepatic fibrosis/hepatic stellate cell activation. The top molecular and cellular functions identified in the RPE were cellular assembly and organization, cellular function and maintenance, protein synthesis, cellular movement and cell–cell signaling and interaction.

**Table 3:** **Pathway Analysis of Differentially Expressed Proteins in Mouse Retina**

Name	# Molecules	p-value
**Top canonical pathways**		
Calcium transport I	2	3.19E-05
Nucleotide excision repair pathway	2	4.17E-04
Platelet homeostasis	2	2.49E-03
NER (nucleotide excision repair, enhanced pathway)	2	2.79E-03
Cellular response to heat stress	2	3.16E-03
**Upstream regulators**		
UBE2W	1	1.61E-03
Sulfonylurea	1	1.61E-03
Phenyl butyrate	1	1.61E-03
HIPK2	2	1.70E-03
Marlboro red cigarette smoke extract	1	2.42E-03

Name	# Molecules	p-value range
**Molecular and cellular functions**		
Molecular transport	7	4.99E-02–2.47E-04
Cell death and survival	3	3.57E-02–8.65E-04
Cell cycle	3	3.49E-02–1.73E-03
Cellular development	5	3.99E-02–1.73E-03
DNA replication, recombination, and repair	4	2.90E-02–1.73E-03

Top DE Proteins Increased (Females vs Male Pool)	Top DE Proteins Decreased (Females vs Male Pool)
LSM7 (1.256)	ATP2A1 (− 1.730)
GSTA3 (1.185)	KRT10 (− 1.509)
HID1 (1.146)	DGUOK (− 1.266)
SELENBP1 (1.106)	SRPRA (− 1.211)
HBA1/HBA2 (1.088)	FBXO22 (− 1.194)
ATP2B4 (1.086)	KYAT1 (− 1.189)
PRUNE1 (1.076)	TPRKB (− 1.160)
SUPT6H (1.067)	HSPA14 (− 1.156)
RRAGA (1.028)	XPO7 (− 1.091)
PFDN2 (1.009)	POLR2B (− 1.064)

**Table 4:** **Pathway Analysis of Differentially Expressed Proteins in Mouse RPE**

Name	# Molecules	p-value
**Top canonical pathways**		
Calcium signaling	9	2.16E-09
Dilated cardiomyopathy signaling pathway	8	2.25E-09
Striated muscle contraction	5	2.13E-08
Actin cytoskeleton signaling	8	1.00E-07
Hepatic fibrosis/hepatic stellate cell activation	7	3.53E-07
Upstream regulators		
PHA-666859	6	1.43E-13
Sesaminol	7	5.93E-12
MAF	7	1.97E-10
Celf1	6	6.41E-10
MEF2C	8	4.05E-09

Name	# Molecules	p-value range
**Molecular and cellular functions **		
Cellular assembly and organization	29	2.32E-02–2.55E-09
Cellular function and maintenance	36	2.49E-02–2.17E-06
Protein synthesis	16	2.49E-02–2.17E-06
Cellular movement	23	2.21E-02–6.68E-06
Cell-to-cell signaling and interaction	21	2.32E-02–3.29E-05

Top DE Proteins Increased in Females vs Male Pool	Top DE Proteins Decreased in Females vs Male Pool
MYH7B (+1.526)	SERPINA1 (− 2.493)
CASQ2 (+1.327)	MUG1/MUG2 (− 1.574)
MYL10 (+1.312)	SERPINA3 (− 1.325)
CRYGC (+1.308)	LAMC1 (− 1.302)
MYOZ2 (+1.308)	TTR (− 1.290)
CRYAA (+1.262)	ACE (− 1.270)
AMPH (+1.252)	MYH4 (− 1.263)
MYL2 (+1.238)	SYNJ1 (− 1.245)
MB (+1.225)	COL1A1 (− 1.237)
MYH3 (+1.219)	DSG1 (− 1.225)

### Enriched GO analysis in the retina and RPE of male and female C57BL6 mice

**Fig. 2:**
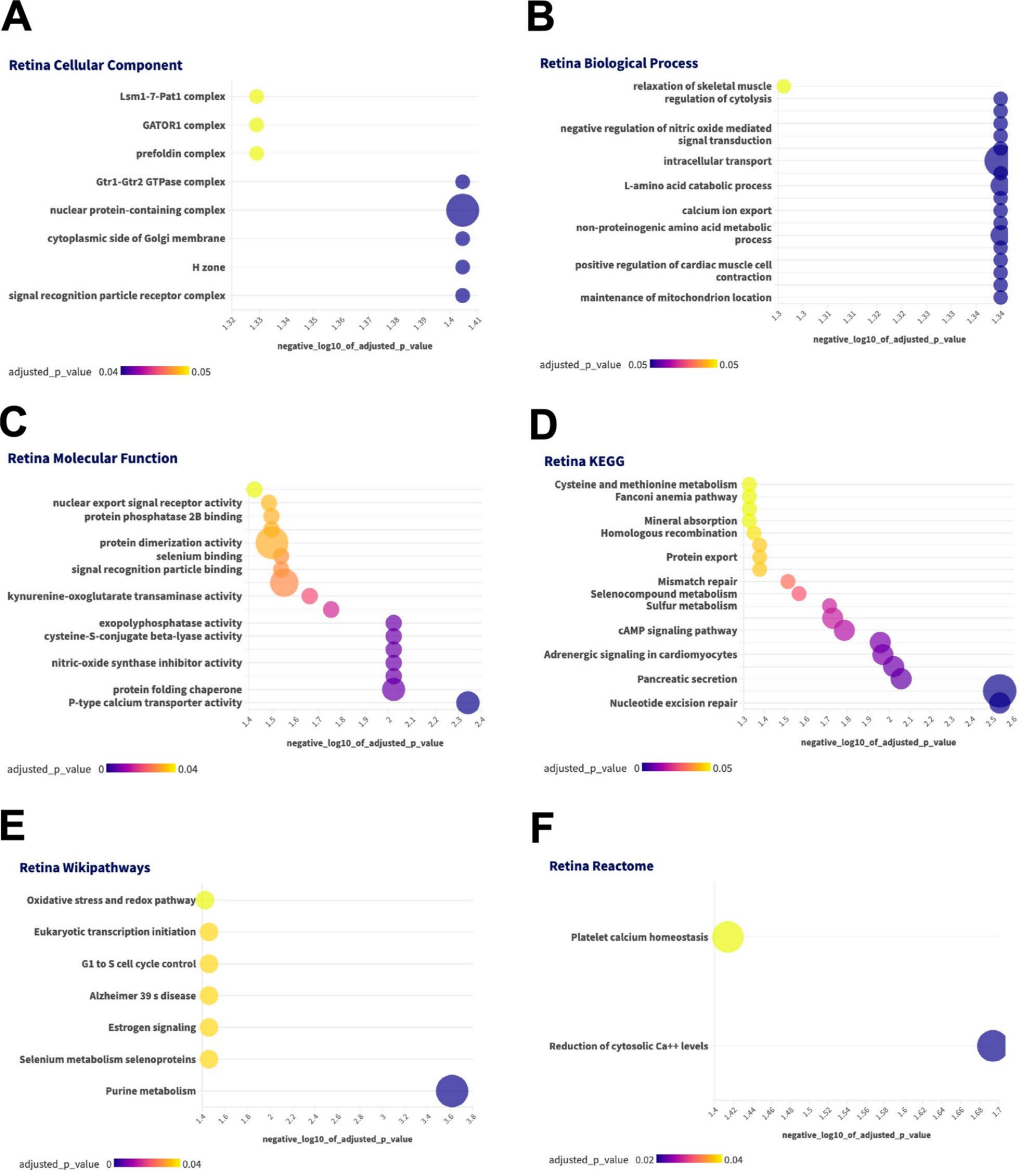
Enrichment Analysis for Retina Proteins. A-C-GO terms. D-KEGG, E-Wikipathways, F-Reactome. Color of bubble correlates with adjusted p-value and size of bubble indicates number of differentially expressed peptides that are associated with the GO term or pathway

**Fig. 3:**
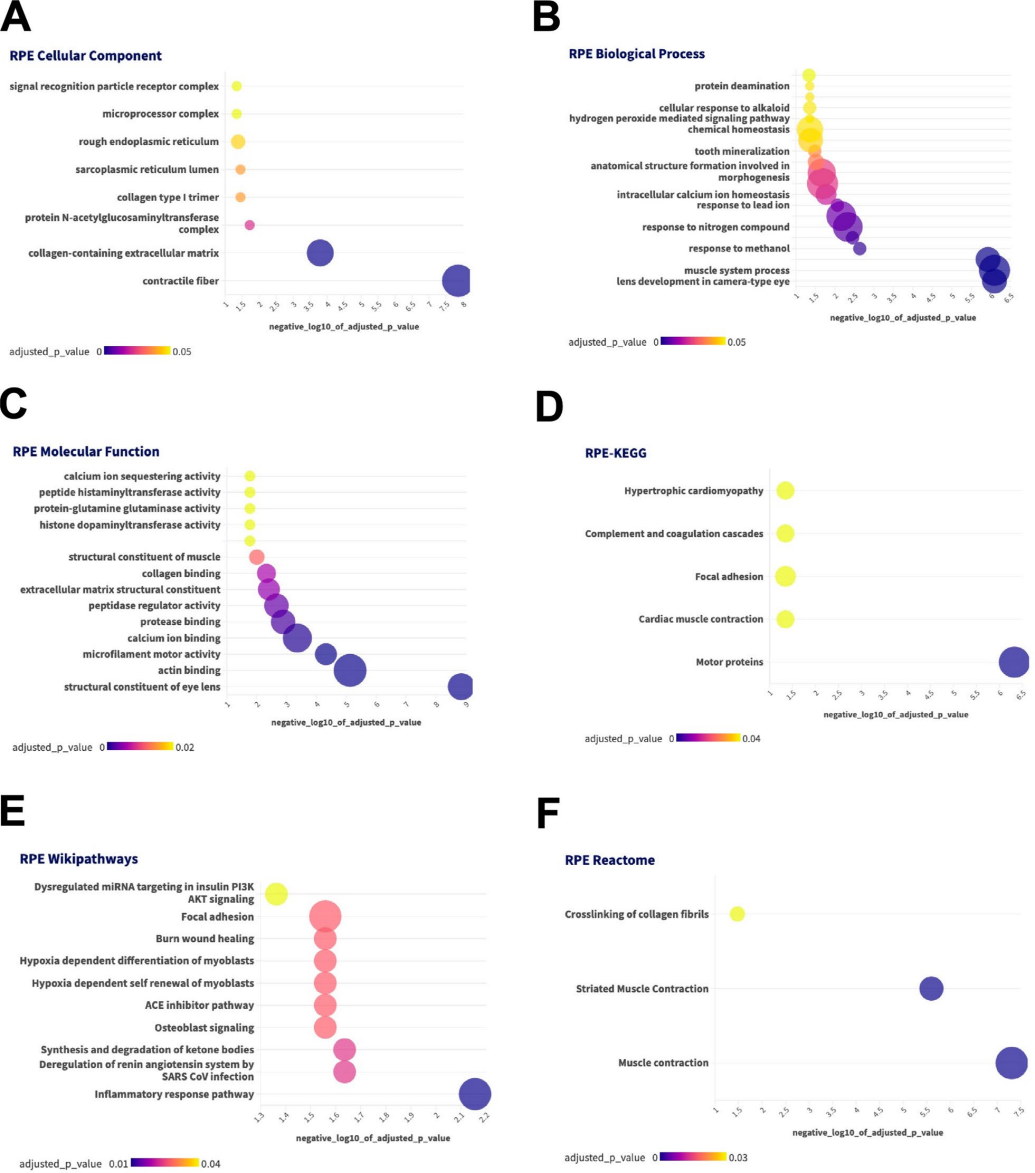
Enrichment Analysis for RPE Proteins. A-C-GO terms. D-KEGG, E-Wikipathways, F-Reactome. Color of bubble correlates with adjusted p-value and size of bubble indicates number of differentially expressed peptides that are associated with the GO term or pathway

Using the differentially expressed proteins and g:Profiler we characterized the GO terms (cellular components, biological processes and molecular functions) as well as KEGG, Reactome and Wikipathways that demonstrated sexual dimorphism in the retina (Fig. 2) and RPE (Fig. 3). The major cellular components associated with the differentially expressed proteins in the retina were nuclear protein containing complex, signal recognition particle receptor complex and cytoplasmic side of Golgi membrane. Intracellular transport, L-amino acid catabolic process, and non-proteinogenic amino acid metabolic processes were the top biological processes and P-type calcium transporter activity and protein folding chaperones were the top molecular functions. Nucleotide excision repair and cAMP signaling pathway (KEGG) and purine metabolism, estrogen signaling, and Alzheimer 39s disease were interesting pathways identified with Wikipathways in the retina.

In the RPE, enrichment analysis revealed that in cellular component, contractile fiber and collagen-containing extracellular matrix were altered while in biological processes, lens development, response to nitrogen compound and intracellular calcium ion homeostasis were differentially regulated. Calcium ion binding, microfilament motor activity, actin binding and structural constituents of the lens were molecular functions identified as differentially expressed between males and females. Motor proteins (KEGG) and inflammatory response pathways (Wikipathways) were also found to be differentially regulated.

## Discussion

In this study, an untargeted quantitative proteomics-based approach was applied to identify baseline sex differences in the protein composition of the RPE and retina in mice. While there was some overlap between the sexual dimorphism in pathway analysis seen in the retina and RPE, there were also unique pathways that were regulated, especially in the RPE. This work serves as a unique resource for the scientific community by defining potential gender differences in the proteome of male and female mouse retina and RPE. Changes in the proteome of the RPE or retina have been examined previously in the context of AMD progression, oxidative stress and diabetic retinopathy [24–35]. However, the normative commonalities and differences in the mouse male and female retina and RPE proteome have not been reported. This data can help future experimental design and interpretation.

A unified theory of the origins of sex differences in tissues was proposed in 2009 [36], which suggests that all ontogenetic sex differences in phenotype are a consequence of the effects of sex chromosome genes. One of these is Sry which controls the sexual differentiation of the gonads and regulates secretion of gonadal hormones (testosterone and estradiol) which have wide-ranging effects to induce organizational (long-lasting/permanent) or activational (reversible) effects on phenotypes of tissues. The theory suggests that in addition to Sry, other X and Y genes have differential effects on male and female cells due to constitutional sex differences in the copy number and/or parental imprint on these genes. Only 2 of the proteins identified as differentially expressed in the RPE were encoded by genes on the X chromosome.

The results presented here establish the following points regarding sexual dimorphism in mouse retina and RPE. The sex differences in the retina and RPE of mice (1) arise from a variety of cell types and affect many cellular processes and pathways (2) are not a direct consequence of sex chromosome genes and (3) are not necessarily related to hormone effects.

Isobaric tags for relative and absolute quantitation (iTRAQ) make it possible to both identify and quantify proteins and in addition, the ability to multiplex allows for high throughput quantitative proteomic analysis. The use of isobaric tags reduces the experimental variability and normalizes potential errors that can potentially arise from sample preparation, digestion or instrument performance. One possible concern that is present in all proteomic analysis is the potential false positives from tissue contamination. While this remains a possibility, we are confident that the large sample numbers (n = 10) and the stringent criteria for qualification as a differentially expressed protein reduces the probability that this contributed to the differences observed. In addition, we can assume that “contamination” if there is any would be random or consistent in all sample preps and would not be picked up in a differential analysis between two groups of samples as they were all prepared the same day by the same investigator.

Sex-dependent differences and divergences in the transcript profiles of mouse retina have been recently reported [37]. In addition, studies have identified signatures of age and sex differences in paw skin and sciatic nerve of naïve mice [38]. In this study we used mice at a single age (12 weeks) to identify sex-based differences. In the future, it will be important to examine the proteome of the retina and RPE to evaluate sex and aging which will provide critical insights into aging diseases of the eye.

We compared our data with that from three published studies which evaluated sexual dimorphism of the proteome in mouse renal proximal tubule [39], mouse paw skin and sciatic nerve [38] and mouse locus coeruleus neuron soma[40]. We found an overlap in 8 of the differentially expressed RPE proteins (Ace, Ces1c, Col1a1, Krt17, Mug1, Serpina1e, Serpina3k, Srprb) and 2 of the differentially expressed retina proteins (Hba and Kyat1) in at least one or more of the tissues examined. Serpina1e was found to be downregulated in females in renal proximal tubules, SCN, skin as well as RPE. Interestingly, a recent study demonstrated that Serpina1e was significantly different in male and female mice during hepatocarcinogenesis [41]. It is not surprising that there is overlap of no more than 10% of the differentially expressed proteins between tissues, as this is more than likely to be a tissue-specific effect. While we have limited our analysis to differentially expressed proteins, there is a possibility that post-translational modifications of proteins might display sexual dimorphism. Future studies evaluating this will likely yield interesting findings. Additionally, future transcriptome-proteome correlation analysis across species and platforms will be important as well.

Since the retina is a complex tissue comprising different cell types, the detected protein differences cannot be ascribed to a specific cell type as can be done in single cell transcriptome studies. It is probably only a matter of time before single cell proteomics will become available for analytical studies and integration of multi-omics will allow for a deeper insight into the molecular mechanisms of aging and disease.

### Perspectives and significance

Despite National Institute of Health (NIH) guidelines to focus on sex as a biological variable, most preclinical studies do not perform this with appropriate statistical power. Majority of publications still report experiments with pooled animals of both sexes. The consequences of this approach are poor data reproducibility, artefactual results and poor translational potential. This study serves primarily as a resource for researchers using mouse models to study ocular physiology and pathology and encourages experimental design to include a careful comparison of sex (and possibly age) to improve the reproducibility and appropriate interpretation of experiments.

## Supplementary Information

Additional file 1.

## Data Availability

In addition to the data reported in the manuscript, all raw data files (Dataset MSV000095302) will be available through the Mass Spectrometry Interactive Virtual Environment http://massive.ucsd.edu
