# Supplementary material for: Quantitative proteomic profiling reveals sexual dimorphism in the retina and RPE of C57BL6 mice
Source: Biol Sex Differ. 2024 Oct 30;15:87. doi: 10.1186/s13293-024-00645-9 (PMC11526624; doi:10.1186/s13293-024-00645-9)
Supplement: Supplementary file 1 — Additional file 1. [file 13293_2024_645_MOESM1_ESM.pdf]

A total of 6 iTRAQ labeled batches analyzed by Thermo Exploris Mass Spectrometer (9 fractions each):

Gender-specific pooled
reference samples were prepared for proteomic analyses
by combining equal amounts of
proteolyzed protein from 10 male and 10 female retinas (10 μg/specimen) and from 8 male and
8 female RPE (5 μg/specimen).

batch 1 (retina)			batch 4 (RPE)	
Sample	iTRAQ tags		Sample	iTRAQ tags
Mp	113		Mp	113
FC1	114		FC1	114
FC2	115		FC2	115
FC3	116		FC3	116
FC4	117		FC4	117
FC5	118		FC5	118
FC6	119		FC6	119
FC7	121		FC7	121
batch 2 (retina)			batch 5 (RPE)	
Sample	iTRAQ tags		Sample	iTRAQ tags
Fp	113		Fp	113
MC1	114		MC1	114
MC2	115		MC2	115
MC3	116		MC3	116
MC4	117		MC4	117
MC5	118		MC5	118
MC6	119		MC6	119
MC7	121		MC7	121
batch 3 (retina)			batch 6 (RPE)	
Sample	iTRAQ tags		Sample	iTRAQ tags
Mp	113		Mp	113
Fp	114		Fp	114
FC8	115		FC8	115
FC9	116		FC9	116
FC10	117		FC10	117
MC8	118		MC8	118
MC9	119		MC9	119
MC10	121		MC10	121

Arranged by tissue (RPE or Retina) and n=10 F vs Male Pool or n=10 M vs Female Pool

Experimental Design Figure				
label	iTRAQ tags	retina batches		RPE batches
FC1/Mp	114/113	batch 1		batch 4
FC2/Mp	115/113	batch 1		batch 4
FC3/Mp	116/113	batch 1		batch 4
FC4/Mp	117/113	batch 1		batch 4
FC5/Mp	118/113	batch 1		batch 4
FC6/Mp	119/113	batch 1		batch 4
FC7/Mp	121/113	batch 1		batch 4
FC8/Mp	115/113	batch 3		batch 6
FC9/Mp	116/113	batch 3		batch 6
FC10/Mp	117/113	batch 3		batch 6
MC1/Fp	114/113	batch 2		batch 5
MC2/Fp	115/113	batch 2		batch 5
MC3/Fp	116/113	batch 2		batch 5
MC4/Fp	117/113	batch 2		batch 5
MC5/Fp	118/113	batch 2		batch 5
MC6/Fp	119/113	batch 2		batch 5
MC7/Fp	121/113	batch 2		batch 5
MC8/Fp	118/114	batch 3		batch 6
MC9/Fp	119/114	batch 3		batch 6
MC10/Fp	121/114	batch 3		batch 6
FC1 Female mouse 1				
Mp: Male Pooled Control				
MC1 Male mouse 1				
Fp: Female Pooled Control				
